# MiR-200b categorizes patients into pancreas cystic lesion subgroups with different malignant potential

**DOI:** 10.1038/s41598-023-47129-1

**Published:** 2023-11-14

**Authors:** Márton Benke, Anikó Zeöld, Ágnes Kittel, Delaram Khamari, István Hritz, Miklós Horváth, Bánk Keczer, Katalin Borka, Ákos Szücs, Zoltán Wiener

**Affiliations:** 1 Department of Surgery, Transplantation and Gastroenterology, Semmelweis University, Budapest, Hungary; 2 Department of Genetics, Cell and Immunobiology, Semmelweis University, Budapest, Hungary; 3 HUN-REN Institute of Experimental Medicine, Budapest, Hungary; 4 Department of Genetics, Cell and Immunobiology, and HUN-REN-SU Translational Extracellular Vesicle Research Group, Semmelweis University, Budapest, Hungary; 5 Department of Pathology, Forensic and Insurance Medicine, Semmelweis University, Budapest, Hungary

**Keywords:** Cancer, Gastroenterology, Medical research

## Abstract

Extracellular vesicles (EV) carry their cargo in a membrane protected form, however, their value in early diagnostics is not well known. Although pancreatic cysts are heterogeneous, they can be clustered into the larger groups of pseudocysts (PC), and serous and mucinous pancreatic cystic neoplasms (S-PCN and M-PCN, respectively). In contrast to PCs and S-PCNs, M-PCNs may progress to malignant pancreatic cancers. Since current diagnostic tools do not meet the criteria of high sensitivity and specificity, novel methods are urgently needed to differentiate M-PCNs from other cysts. We show that cyst fluid is a rich source of EVs that are positive and negative for the EV markers CD63 and CD81, respectively. Whereas we found no difference in the EV number when comparing M-PCN with other pancreatic cysts, our EV-based biomarker identification showed that EVs from M-PCNs had a higher level of miR-200b. We also prove that not only EV-derived, but also total cyst fluid miR-200b discriminates patients with M-PCN from other pancreatic cysts with a higher sensitivity and specificity compared to other diagnostic methods, providing the possibility for clinical applications. Our results show that measuring miR-200b in cyst fluid-derived EVs or from cyst fluid may be clinically important in categorizing patients.

## Introduction

With the emergence of high-resolution cross-sectional imaging modalities, a high number of pancreatic cystic lesions are diagnosed incidentally. These lesions consist of the heterogeneous group of pancreatic cystic neoplasms (PCN) and pseudocysts (PC) ^1^. Serous cystadenomas are serous pancreatic cystic neoplasias (S-PCN), and similar to PCs, they have a very low chance to develop into malignant disease ^2,3^. Other PCNs consist of borderline malignant and premalignant lesions, such as intraductal papillary mucinous neoplasms and mucinous cystic neoplasms. Since these two subtypes are clinically the most significant due to their potential malignant behavior, we considered them as one group in our work (mucinous PCN, M-PCN) ^2,4,5^.

Most PCNs are found on computed tomography (CT) images. Since PCNs can only be differentiated with a 40–81% sensitivity on CTs ^6–8^, magnetic resonance imaging (MRI) and magnetic resonance cholangiopancreatography (MRCP) are often performed to supplement the diagnosis. However, a combined imaging method is usually required as it has a significantly higher accuracy ^6,9^. Endoscopic ultrasound (EUS) is another diagnostic modality, recommended in cases where the CT and MRI are not decisive or where radiological or clinical features suggest malignant potential ^2^. In addition, fluid samples from cystic lesions can also be analyzed for carcinoembryonic antigen (CEA) level ^10^. According to Brugge et al., CEA levels from cyst fluid samples above 192 ng/mL can refer to lesions of mucinous nature, with a sensitivity of 75% and specificity of 83.6% ^10^. Similar to the cyst fluid, cytology obtained from EUS is useful only as a complementary tool in diagnosing M-PCNs ^11,12^.

The heterogeneous nature of the pancreatic cystic lesions makes it very difficult to avoid excessive surgical treatment of benign cases. Patients with pancreatic cysts, but without history of pancreatitis have an increased relative risk to develop pancreatic cancer, such as pancreatic ductal adenocarcinoma (PDAC) ^13^. In these cases, timely surgical treatment is essential. However, the surgery to remove these lesions is very invasive and it is associated with high morbidity and mortality rates. Unfortunately, the strict interpretation of clinical parameters suggesting relative or absolute indications for surgery vary widely and they are not objective enough. In addition, some of them can only be obtained by EUS morphology analysis which is an operator dependent and frequently subjective modality ^14^. Thus, indications for pancreatic resections based only on these criteria are not accurate, and they are used due to the lack of other more reliable options ^15^.

Extracellular vesicles (EV) transport biologically important molecules, such as miRNAs, and they are considered as novel promising biomarkers in cancers and in other diseases ^16,17^. Since isolating EV subtypes based on their cellular origin is difficult, they are often clustered according to their size ^18^. This results in large (lEV), medium sized (mEV) and small EVs (sEV) when using different centrifugation and ultracentrifugation speeds ^19^.

Recently, we and others have isolated EVs from the peripheral blood of PDAC patients and the miRNA cargo analysis of EVs has been carried out ^20–22^. Interestingly, these studies led to the identification of different promising EV cargo components as markers of PDAC. In addition, a recent elegant study used EVs to predict the malignant potential of IPMNs. Yang et al. used peripheral blood and they identified MUC5AC protein as an EV cargo component with a predictive power for the malignant progression of IPMNs ^23^.

Since malignant conversion of M-PCN, but not of S-PCN or PC may occur, discriminating M-PCN from all other groups (not-M, containing S-PCN and PC) may be clinically relevant. However, an objective and reliable parameter is missing to help the clinical decision making process. To address this question, we analyzed cyst fluid samples and their EV content. We carried out an EV-based biomarker search to identify promising candidates that decide whether the pancreatic cyst falls into the category of M-PCNs.

## Results

### Pseudocystic and PCN cyst fluid samples contain CD63+ EVs

**Figure 1:**
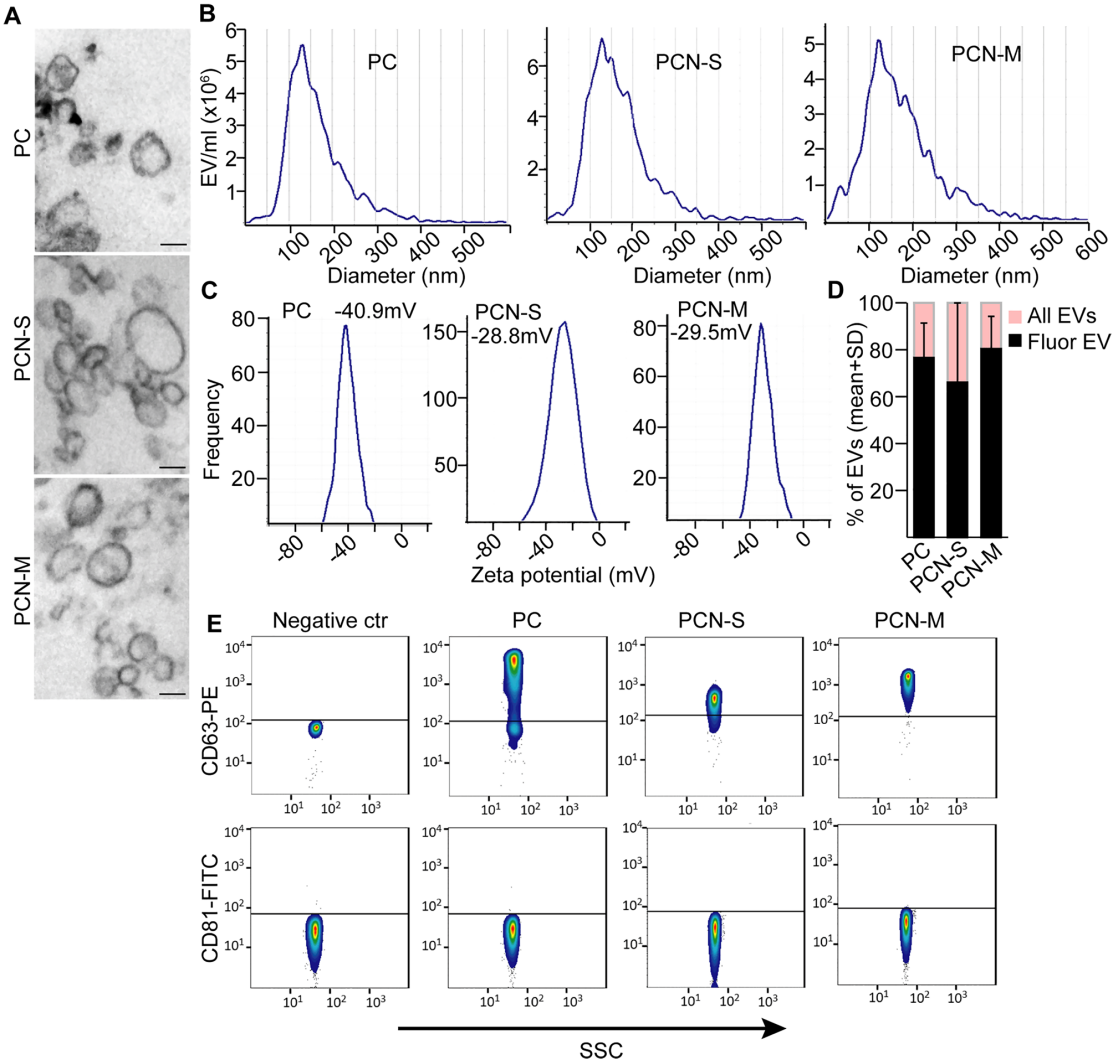
Pancreatic cyst fluid contains EVs. (**A**) Transmission electron microscopic (TEM) images from the EV-enriched ultracentrifuged pellet isolated from PC, S-PCN or M-PCN patients. (**B**) Nanoparticle tracking analysis (NTA) from the cyst fluid samples of the indicated patients. (**C**) Zeta potentials after removing cells and cell fragments by centrifuging samples at 300*g*. (**D**) The ratio of fluorescent EVs. Total EV number was measured and EVs were visualized in the same sample with a membrane labelling dye with NTA. (**E**) Flow cytometry-based analysis of EVs in cyst fluids. EVs were isolated with anti-CD63 or anti-CD81-coated beads after centrifuging samples at 300*g*, and labelled with anti-CD63 or anti-CD81, respectively. Beads incubated in DMEM were used as negative control. Scale bars: 100 nm.

Since EVs and their cargo are considered as promising diagnostic tools, we characterized EVs in PC, S-PCN and M-PCN cyst fluid samples as an initial step. Patients were classified based on cytological results, CEA level, EUS morphology and imaging methods in multidisciplinary clinical panel discussions (for patient data see “Methods”). After classifying patients, we proved the presence of EVs in PC, S-PCN and M-PCN samples by transmission electron microscopy (TEM) (Fig. 1A) and Nanoparticle Tracking Analysis (NTA) that is a widely used method to determine the size distribution and concentration of EVs (Fig. 1B). In addition, the high absolute value (> 20 mV) ^24^ of zeta potential indicated that even after storage the EV suspension was stable and lacked aggregation ^25^ (Fig. 1C). In addition, applying a membrane labelling dye and NTA showed that the majority of particles had an intact membrane (Fig. 1D). CD81 and CD63 are markers of EVs. By applying anti-CD63 or anti-CD81 antibody-coated beads and flow cytometry, we observed that EVs derived from all sample groups were positive for CD63, but, surprisingly, they were negative for CD81 (Fig. 1E). Collectively, we proved the presence of CD63+ EVs in PC, S-PCN and M-PCN.

### miR-200b is present at a higher level in M-PCN-derived EVs compared to EVs isolated from not-M samples

**Figure 2:**
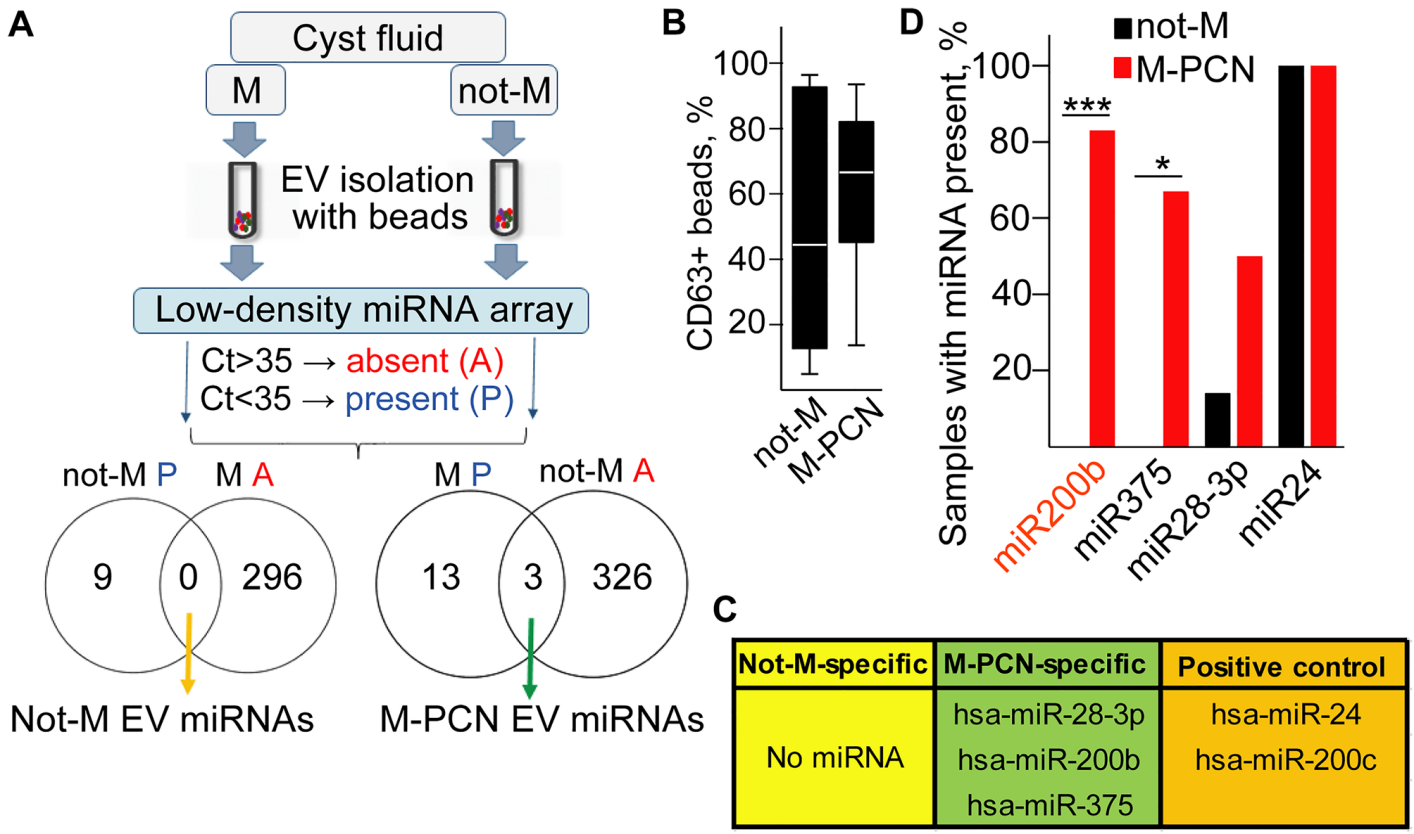
M-PCN-derived EVs carry a specific miRNA set. (**A**) The schematic workflow of the experiments. For details see “Methods”. (**B**) The percentage of CD63+ beads in not-M and M-PCN patients, detected by flow cytometry. (**C**) The M-PCN EV-specific miRNA set and positive control miRNAs. (**D**) The percentage of samples in the discovery group where the indicated members of the M-PCN EV miRNA set were present (Ct < 35). Note that miR-200b showed the largest difference when comparing not-M (n = 7) and M-PCN (n = 6). Chi square test, *p < 0.05 and ***p < 0.001. See also Tables S1 and S2.

Since miRNAs are important components of the EV cargo, we focused on these molecules in our further experiments. Previously we found that isolating EVs with antibody-coated beads resulted in a lower unspecific miRNA background compared to the widely used differential centrifugation and ultracentrifugation or commercially available EV purification kits ^26^. Thus, we applied anti-CD63-coated beads to isolate EVs from cell-free cyst fluids after a low-speed centrifugation, containing all EVs, and we analyzed the level of 377 miRNAs with low-density miRNA arrays. In contrast to S-PCN and PC patients where malignant transformation is extremely rare, M-PCNs may have a malignant potential. Thus, for our miRNA analysis we used the two categories of not-M (S-PCN and PC as a unified group) and M-PCN with the aim to find significant differences in EV cargo composition. We then selected miRNAs that were differentially present between the two groups (for details see “Methods”, Fig. 2A, Table S1). Of note, we used an equal starting sample volume in these studies, and we observed no difference in the proportion of CD63+ EV-containing beads between not-M and M-PCN samples (Fig. 2B). Whereas no EV miRNAs turned out to be specific for the not-M group using stringent selection criteria, we found that miR28-3p, miR-200b, and miR-375 were present in all M-PCNs, and at most in only one not-M sample (Table S1 and Fig. 2C) which we designated as “M-PCN specific EV miRNAs”. In addition, we found that miR-24 and miR-200c were present (Ct < 35) in minimum 12 out of the 13 analyzed samples, raising the possibility that these miRNAs may be used as positive controls (Fig. 2C).

For further verification of our results, we isolated EVs from equal volumes (500 µL) of samples after low-speed centrifugation with anti-CD63-coated beads, and we applied individual assays for the M-PCN specific EV miRNA set (discovery patient cohort). Since we previously found the constant level of miR-24 in PDAC patient-derived EVs ^22^, we selected this miRNA as positive control, and we detected miR-24 in all samples (Table S2). When analyzing samples used for the miRNA array, we observed that out of the M-PCN specific EV miRNAs, EV miR-200b was absent in all not-Ms, but it was present in the vast majority of M-PCNs (Fig. 2D, Table S2).

### No correlation between EV numbers, diagnosis or the clinical types of PCN

**Figure 3:**
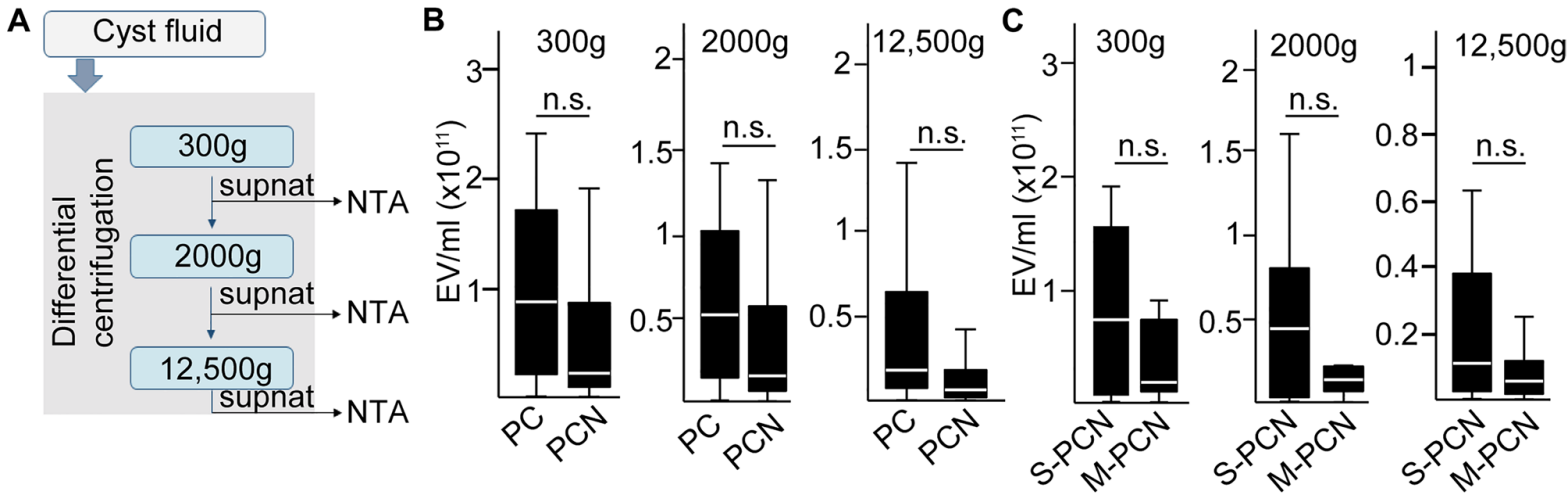
No significant difference in cystic fluid EV numbers between the patient groups. (**A**) The schematic workflow of the experiments. (**B**,**C**) The concentration of EVs in the cyst fluid of (**B**) PC and PCN or (**C**) serous PCN and mucinous PCN patients, measured with NTA. n = 10–20 from the discovery and validation cohorts. p > 0.05 for all comparisons (Mann–Whitney U-test).

We then used another sample set (validation cohort), and we analyzed EV fractions from supernatants of the discovery and validation samples after serial centrifugation steps with NTA (Fig. 3A). This approach allows to measure all EVs after removing cells, EVs without large EVs or only small EVs. Importantly, we found no correlation between the number of EVs and the diagnosis (PC or PCN, Fig. 3B) independently of the size ranges of the analyzed EVs. Similarly, no significant change in EV number was observed when comparing S-PCN and M-PCN (Fig. 3C), proving the lack of correlation between EV numbers and patient groups.

### The level of EV miR-200b cargo discriminates M-PCNs from not-M pancreatic cysts in a validation patient cohort

**Figure 4:**
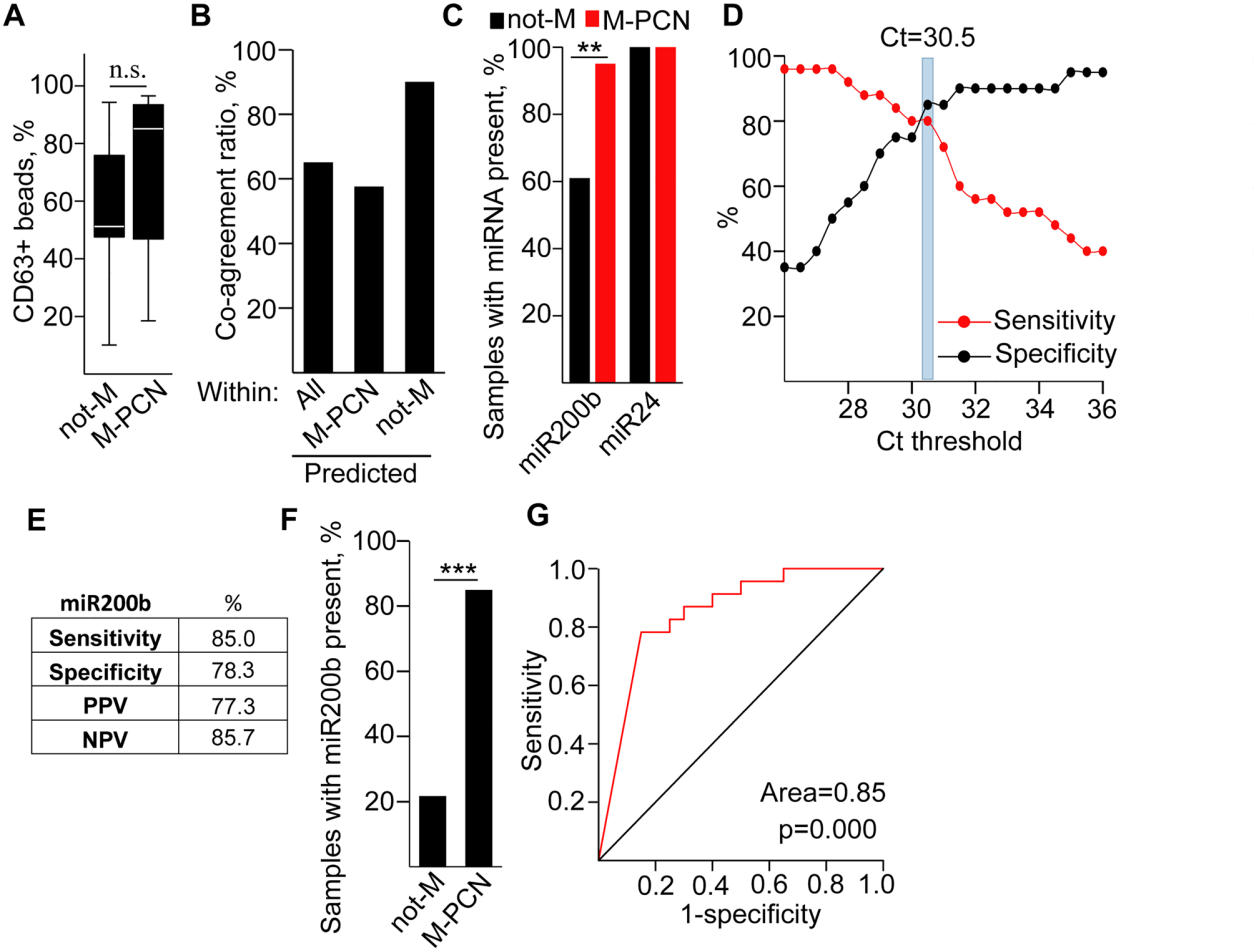
The miR-200b level in EVs from equal volumes of cystic fluid samples categorizes patients of the validation cohort into not-M and M-PCN groups. (**A**) Percentage of CD63+ beads in M-PCN and not-M, detected by flow cytometry. (**B**) Ratio of samples showing a co-agreement between miR-200b analysis and the categorization by the multidisciplinary panel. Note that samples were first classified based on the presence (Ct < 35) or absence (Ct > 35) of miR-200b into M-PCN (n = 33) and not-M (n = 10) groups, and the proportion of samples with the same clinical diagnosis is illustrated. (**C**) Percentage of samples with miR-200b present flag within the not-M (n = 23) and M-PCN (n = 20) groups (classified by the multidisciplinary clinical panel). (**D**) Depicting the specificity and sensitivity as a function of the Ct threshold for discriminating between the present and absent flags. (**E**) Sensitivity, specificity, positive (PPV) and negative predictive value (NPV) for cyst fluid EV miR-200b level when using a Ct = 30.5 threshold for present and absent flags (comparing M-PCN and not-M). (**F**) The percentage of samples with miR-200b present in cyst fluid (Ct < 30.5, n = 23 and n = 20 for not-M and M-PCN, respectively). (**G**) ROC curve analysis of miR-200b Ct values. Note that the area of 0.85 is considered as a good category for the tests. Mann–Whitney U-test (**A**) or Chi-square test (**C**,**F**) were used with n.s.: p > 0.05, **p < 0.01 and ***p < 0.005. See also Table S3.

Based on our results obtained with miRNA arrays, strengthened with the discovery patient set, we focused on miR-200b. Similarly to the discovery cohort, we first applied an equal amount of cyst fluid for EV isolation with antibody-coated beads from validation samples, and we used a cutoff value of Ct = 35 for miR-200b with present or absent labels. Similarly to the discovery set, we found no difference in the percentage of positive anti-CD63 coated beads when they were incubated in not-M or in M-PCN cyst fluids (Fig. 4A). Interestingly, the EV miR-200b based classification resulted in a > 65% co-agreement with the categorization by the multidisciplinary clinical panel, and we observed similar values when focusing only on samples predicted to be not-M or M-PCN by EV miR-200b (Fig. 4B). In line with these data, samples with miR-200b present flag (Ct < 35) accumulated in the M group (Fig. 4C, Table S3). Thus, the validating sample set recapitulated results from the discovery cohort.

Since we observed that many S-PCN samples within the not-M group produced a Ct < 35 value and a presence flag in the validation cohort (Table S3), we analyzed the sensitivity and specificity for EV miR-200b levels to find the optimal Ct threshold value to discriminate M-PCN and not-M. When applying a binary system with “present” Ct < 30.5 and “absent” above this cutoff value, we found that sensitivity and specificity were higher than 78%, thus, we applied this threshold (Fig. 4D,E). Interestingly, we observed that miR-200b was significantly more frequently present in EVs isolated from M-PCN compared to not-M (Fig. 4F). Of note, ROC curve analysis showed that EV miR-200b can be used as a good test (area > 0.8) to discriminate M-PCN and not-M (Fig. 4G). We observed similar results when repeating experiments with an equal starting amount of EVs, determined with NTA, where we found a higher frequency of samples with the present flag (Ct < 30.5) of miR-200b in M-PCN compared to not-M of the validation cohort (Table S4).

**Figure 5:**
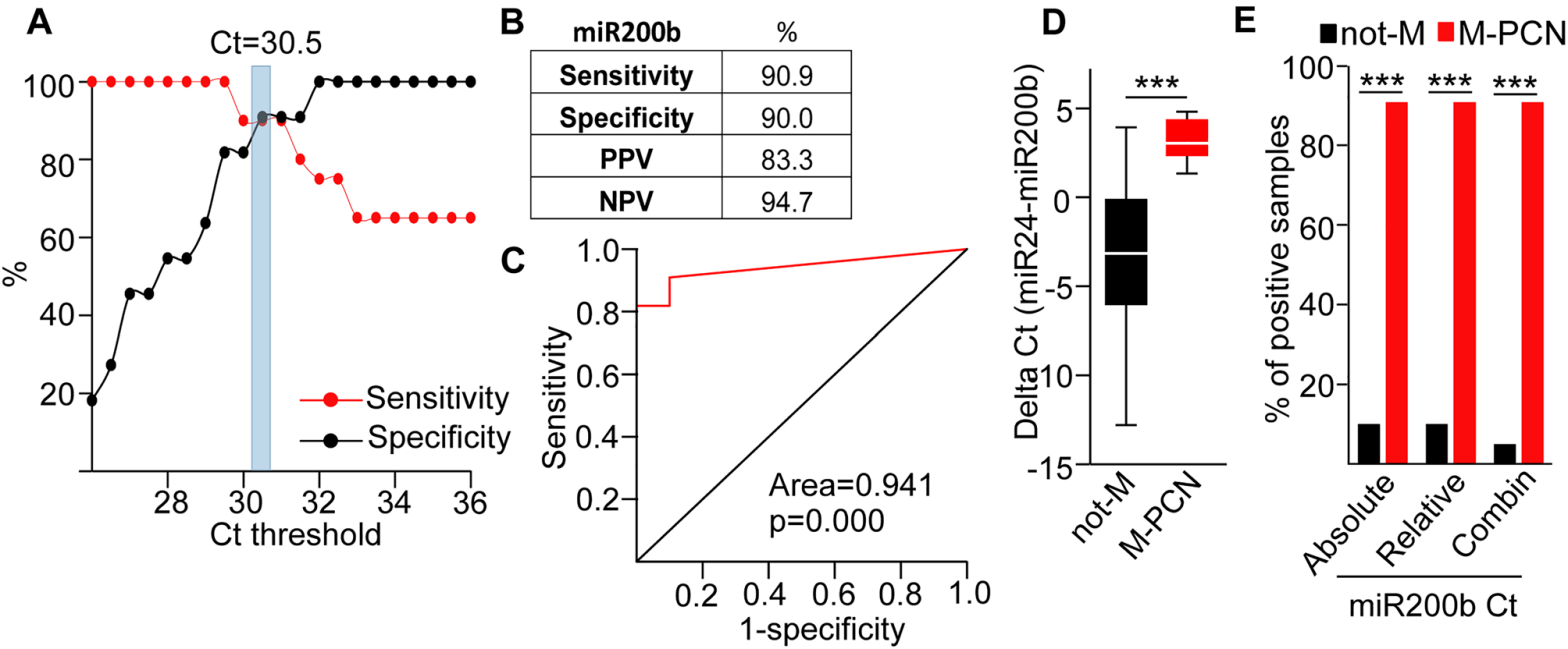
Measuring EV miR-200b in cystic fluid samples normalized to equal RNA amounts categorizes patients into not-M and M-PCN groups. (**A**) The specificity and sensitivity as a function of the Ct threshold for discriminating between the present and absent flags. (**B**) Sensitivity, specificity, positive (PPV) and negative predictive value (NPV) for cyst fluid EV miR-200b level (threshold for absent and present: Ct = 30.5). (**C**) ROC curve analysis of miR-200b Ct values. (**D**) The distribution of the relative miR-200b Ct values between the indicated patient groups. Relative Ct values were determined as the difference between Ct for miR-24 and for miR-200b. (**E**) The percentage of samples with present flags in not-M and M-PCN groups. Ct < 30.5 for miR-200b and ΔCt (miR-24-miR-200b) > 2 were used for absolute and relative quantitation thresholds, respectively. Note that the combination of these two parameters (Ct < 30.5 and ΔCt > 2) had the best discriminating effect on M-PCNs. n = 20 for not-M and n = 11 for M-PCN. Mann–Whitney U-test (**D**) and Chi-square test (**E**) were used with ***p < 0.001. See also Table S5.

Thus, using a standard volume of samples or an equal amount of EVs, combined with a standard protocol for EV isolation and cargo characterization, including detecting miR-24 in the EV preparate as a positive control may be clinically relevant. To further improve our detection method, we used an equal RNA amount from EV preparates (Table S5) and we repeated categorization of not-M and M-PCN samples. When applying absolute quantitation of miR-200b with a Ct = 30.5 threshold, we observed a > 90% sensitivity, specificity and negative predictive value (NPV), and a > 80% positive predictive value (PPV) (Fig. 5A,B). In addition, ROC curve analysis showed the successful discrimination between M-PCN and not-M (Fig. 5C). As a further analysis, we also calculated the relative Ct value of miR-200b compared to the positive control miR-24, and we observed a significant difference between the not-M and M-PCN groups (Fig. 5D). Importantly, combining the absolute and relative Ct values with a threshold of Ct < 30.5 for miR-200b and Ct > 2 for the difference of miR-24 and miR-200b to define the M-PCN group, resulted in a further improvement in separating not-Ms and M-PCNs (Table S5, Fig. 5E). Collectively, all these results suggested that determining the combined absolute and relative miR-200b levels in EV samples that have been normalized to total RNA amount from pancreatic cyst fluids may be a useful parameter to classify patients into M-PCN or not-M groups. Importantly, these two groups have different risks for malignancy.

We then selected operated patients with known histological results from both cohorts, and we re-analyzed their volume-normalized EV-based miR-24 and miR-200b data (Table S6). As expected, they all belonged to the M group. Interestingly, we found a tendency to higher miR-200b levels (normalized to miR-24) in patients with a malignant histological result compared to the benign samples (Table S6), although the difference was not significant probably due to the low sample number. Thus, cyst fluid EV-based miR-200b level may also indicate patients of the M group with malignancy, however, further studies should confirm this finding with a larger sample size.

### Total miR-200b level in pancreatic cyst fluid samples has a discriminating potential between M-PCNs and not-M samples

**Figure 6:**
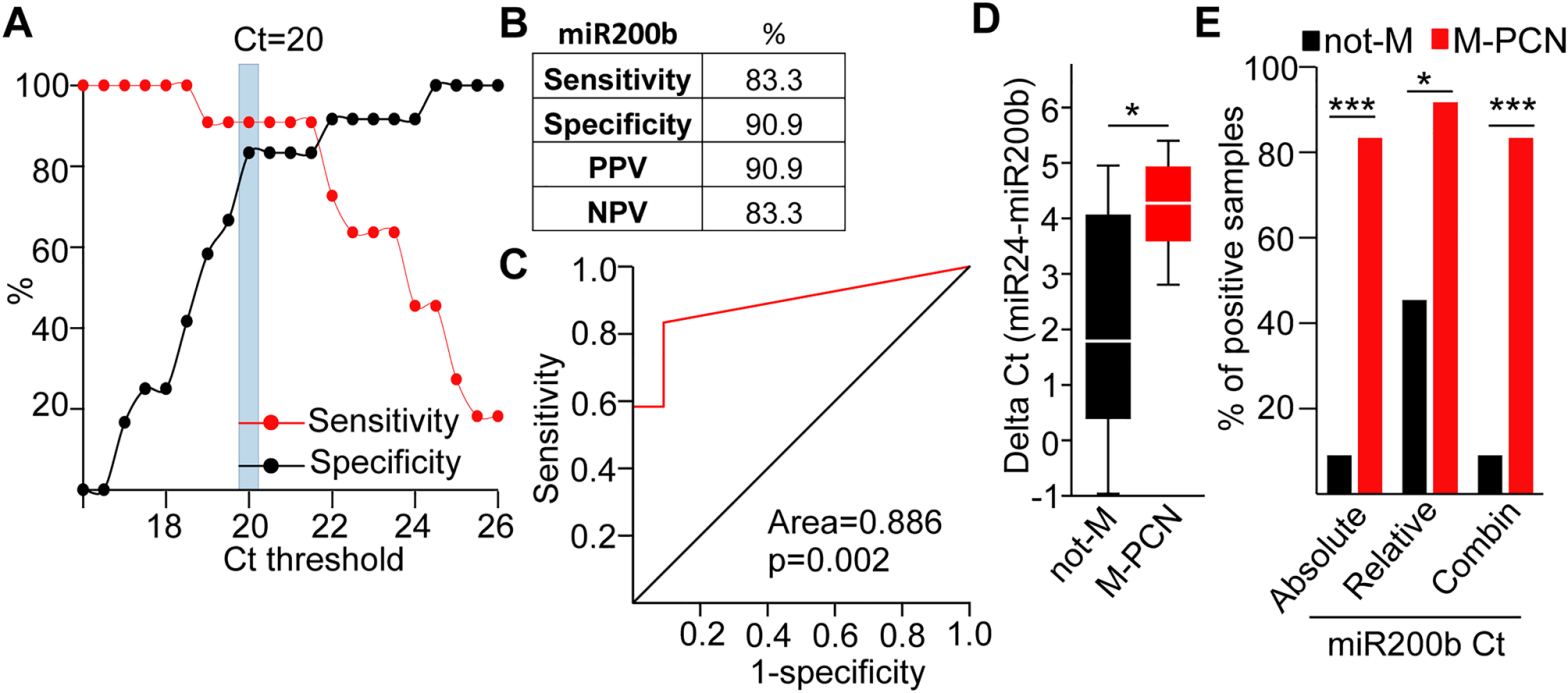
miR-200b measured from total cyst fluid discriminates not-M and M-PCN samples. (**A**) Determining the optimal Ct threshold for discriminating between the present and absent flags. Note that Ct = 20–21 has the best performance. (**B**) Sensitivity, specificity, positive (PPV) and negative predictive value (NPV) (threshold for absent and present: Ct = 20). (**C**) ROC curve analysis of miR-200b Ct values. (**D**) The distribution of the relative miR-200b Ct values (difference between Ct for miR-24 and miR-200b) for the indicated patient groups. (**E**) The percentage of samples with present flags in the not-M and M-PCN groups. Ct < 20 for miR-200b and ΔCt (miR-24-miR-200b) > 2 were used for absolute and relative quantitation cutoffs, respectively. n = 11 for not-M and n = 12 for M-PCN. Mann–Whitney U-test (**D**) and Chi-square test (**E**) were used with *p < 0.05 and ***p < 0.001. See also Table S7.

The clinical handling of EVs is difficult, and only few studies compared the potential application of EV cargo analysis and miRNA levels in total body fluid samples in early diagnostics. To test this possibility, we carried out miR-200b and miR-24 analysis, normalized to total RNA amount, from cystic fluid samples as well (Table S7). By using the same approach as for EV miRNA cargo, we observed that absolute quantitation of miR-200b with Ct < 20 present and Ct > 20 absent flags had a high sensitivity, specificity, NPV and PPV. Furthermore, ROC analysis showed the discriminating power between not-Ms and M-PCNs (Fig. 6A–C). In addition, we observed a difference in the relative Ct of miR-200b, normalized to miR-24, between the two patient groups (Fig. 6D). Importantly, the combination of Ct < 20 absolute and Ct > 2 relative miR-200b values to mark M-PCN patients performed similarly in discriminating M-PCNs and not-Ms compared to the absolute values alone (Table S7, Fig. 6E).

### The level of miR-200b shows the best correlation with results from EUS

**Figure 7:**
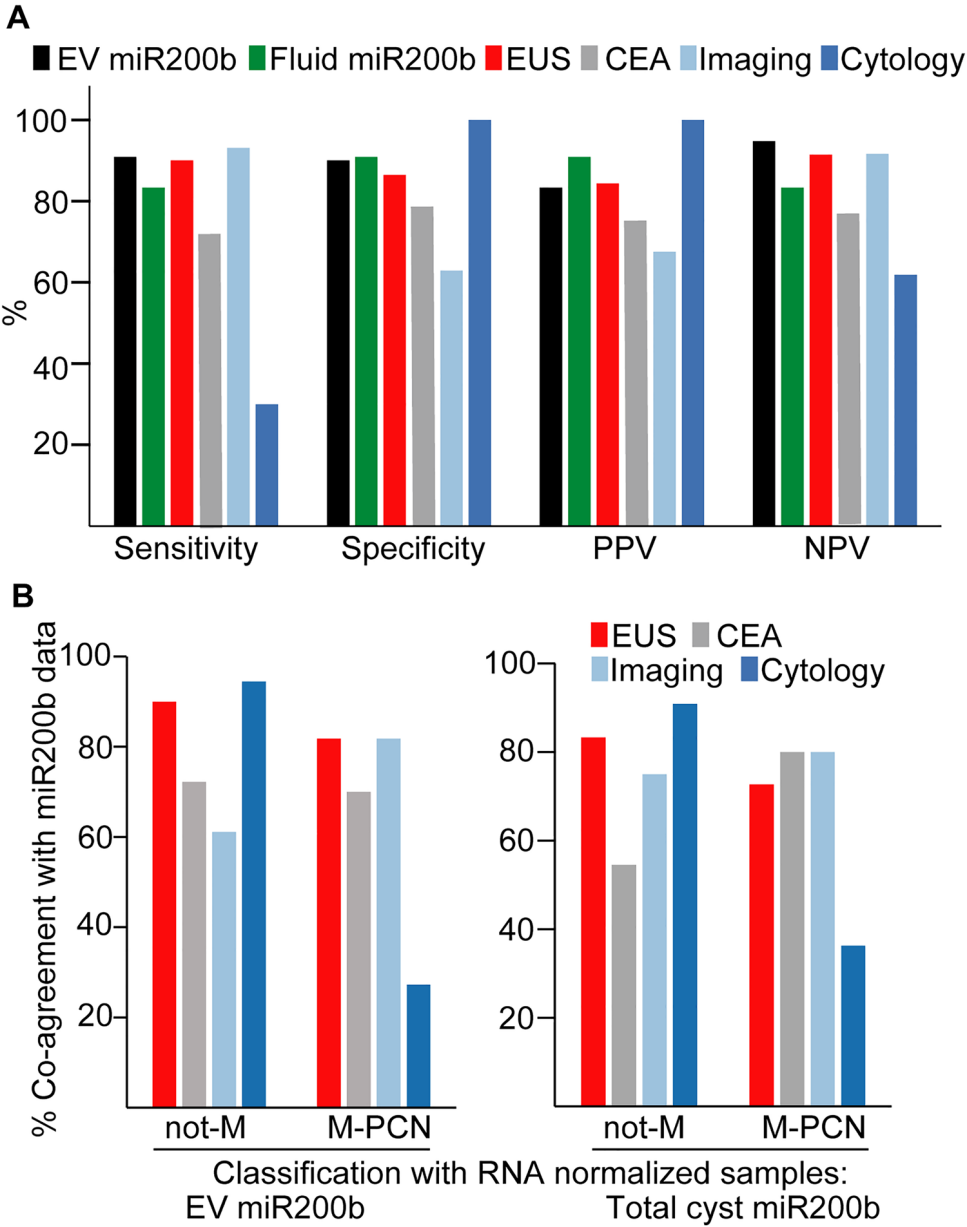
miR-200b level in cyst fluid and EUS morphology perform better than other diagnostic methods. (**A**) Sensitivity, specificity, PPV and NPV for the indicated methods. (**B**) Percentage of cases with co-agreement between miR-200b level and other methods. Note that RNA-normalized miR-200b analyzed with the combination of absolute and relative Ct values (see Figs. 5 and 6) were classified into not-M and M-PCN groups. Within these categories, the prediction with other methods (not-M or M-PCN) was determined. Data were obtained from total cyst fluid or from isolated EVs. Note the high co-agreement with the EUS method. See also Table S8 for the number of samples.

The clear clinical diagnosis is largely inhibited by the frequently contradicting results from EUS morphology, cytologic results, imaging and CEA. Thus, we next compared the performance of these methods with cyst fluid total and EV miR-200b levels in discriminating between the not-M and M-PCN groups. Since cystic fluid samples above 192 ng/mL CEA levels have been found to refer to lesions with mucinous features ^10^, we considered this value as threshold. Interestingly, miR-200b level and EUS morphology had a > 80% for sensitivity, specificity, PPV and NPV in our patient cohort. In contrast, imaging, cytology or CEA reached only a < 80% value in at least one of these parameters (Fig. 7A). When categorizing samples based on EV or total cyst fluid miR-200b levels into not-M and M-PCN, we found the best co-agreement with EUS morphology (Fig. 7B). Thus, both total cyst fluid and EV miR-200b cargo levels showed the best correlation with EUS morphology among the traditional diagnostic methods for M-PCN and not-M patients.

## Discussion

Since EVs may be novel diagnostic tools in several diseases, we decided to carry out an EV-based search for biomarkers to distinguish M-PCNs. We show here that PC and PCN fluid samples contain CD63+ but not CD81+ EVs. Interestingly, we found no correlation between the concentration of EVs and patient categories (PC or PCN, M-PCN or not-M containing serous PCN and PC patients). We also provide evidence that M-PCN-derived EVs share a common miRNA set, containing miR-28-3p, miR-200b, and miR-375, showing the different cargo composition of EVs among patient groups. When comparing M-PCN with not-M, we found that miR-200b was specific to M-PCN-derived EVs. In addition, we also prove that not only EV, but also total cyst fluid derived miR-200b is suitable to classify patients into M-PCN and not-M groups, raising the possibility of developing a test that may be easier implemented in the clinic. Of note, this miRNA performs better in both sensitivity, specificity, positive and negative predictive value compared to some other classical diagnostic methods, such as CEA or imaging techniques.

EVs are present in most body fluids, thus, they are considered an easily available source of disease-specific molecules. In our work we provide evidence that pancreatic cyst fluid contains EVs. Interestingly, we found that these EVs showed positivity for CD63, however, we could not detect CD81 on their surface. This is in line with our previous results showing high abundancy of CD63+ but not CD81+ EVs in pancreatic juice ^27^. Furthermore, we previously reported that blood from both normal and pancreatic ductal adenocarcinoma (PDAC) patients had CD63+/CD81− EVs ^22^. In addition, a recent work published that the majority of blood EVs had only CD63. Interestingly, CD63+ EVs were enriched in serum, while CD81+ EVs were rare in both plasma and serum ^28^. Whether EVs with CD63 or CD81 are of different cellular origin and this difference has a functional importance as well, is at present not clear.

One of the most important cargo components of EVs are miRNAs. The miRNA content of EVs has already been extensively studied in many diseases, such as in pancreatic cancers ^20–22^. Although a recent work analyzed EV cargo in IPMN, a subgroup of M-PCN, they focused on proteins from blood-derived EVs ^23^. In contrast, we studied directly the miRNA cargo of pancreatic cystic fluid-derived EVs to search for potential novel biomarkers, and we found that some miRNAs were present at different levels in EVs from different patient groups. Importantly, our study is the first to show a correlation between the level of miR-200b and the mucinous nature of PCNs in cyst fluid. Members of the miR-200 family (200a, b, c, 429, 141) share the same seed sequence and are fine tuners of epithelial-mesenchymal transition (EMT), a key process in invasion ^29,30^. By targeting specific transcription factors ^31,32^, they are involved in the suppression of EMT. The controversial role of this miRNA family is clearly illustrated by the fact that they may facilitate metastasis outgrowth by enhanced mesenchymal-epithelial transition (MET). MiR-200b is also critical in regulating the level of transcription factors involved in the proliferation of cancer cells ^33^. Some studies also suggested that serum EV miR-200b may be a biomarker for diagnosing PDAC ^34^. Although the role of miR-200b in M-PCN cyst fluid is not known, our studies prove that this miRNA is not specific for PDAC and it is already present in pancreatic cysts with the potential to malignancy. In line with these data, we previously found that some miRNAs characteristic for PDAC patient-derived EVs may already be present in chronic pancreatitis, too ^22^. In addition, Lee et al. similarly identified miR-200b as a differentially expressed miRNA when comparing M-PCNs with other pancreatic cysts ^35^. Although their results co-agree to our findings, however, this study used resected specimens with a limited potential for pre-operative diagnosis. Collectively, all these data suggest that miR-200b may be an appropriate biomarker for the prediction of PCN subtypes that may have a malignant potential, however, further validations are still required.

Previous analyses of candidate miRNAs in pancreatic cysts reported that miR-21 and miR-155 were upregulated in IPMN ^36,37^. Others reported that miR-10a had a higher level in invasive pancreatic cysts ^38^. Interestingly, our analysis did not identify them as different miRNAs between M-PCN and not-M EVs. This discrepancy may be explained by the fact that, in contrast to our experiments where we applied an EV-based screen for potential biomarkers, these studies focused primarily on tissue samples and they used different patient groups. Importantly however, we proved that miR-200b level discriminates not-M and M-PCN patients not only when measured from EVs, but also from total cyst fluid, although with a worse sensitivity and negative predictive value (NPV). Since handling EVs in routine diagnostics is still challenging, analyzing cyst fluid may be easier in the clinic. However, future studies should involve a larger number of samples to decide whether the better sensitivity and NPV of EV-based miR-200b analysis compared to testing total cyst fluid samples has a significant clinical value.

In contrast to not-M, M-PCN patients have a risk for malignancy, thus, diagnosing patients with M-PCN is extremely important in the clinical practice ^39^. Of note, we found here that measuring the miR-200b level in cystic fluid has a better specificity, sensitivity, positive and negative predictive value compared to many other classical diagnostic methods, such as imaging or CEA ^40^. When using an optimized analysis for miR-200b, all these parameters were higher than 83%. In contrast, the concentration of CEA in the cystic fluids with a cutoff value of 192 ng/ml can differentiate between mucinous and serous PCN with a sensitivity of 75% ^10^. Similarly, the accuracy of EUS morphology and imaging modalities has a wide range of efficiency for differentiating PCN subtypes ^41,42^. Cytological examination of the cyst fluid is very specific (83–100%), but very insensitive (27–48%). Thus, all these methods are inaccurate in predicting lesions with a malignant potential ^6^.

Collectively, we provide here evidence that pancreatic cyst fluid is a rich source of EVs. Interestingly, our EV-based screen showed that EVs from M-PCNs have a higher level of miR-200b that discriminates patients with M-PCN from other pancreatic cysts. Of note, miR-200b in the total cyst fluid has a good discriminating value as well, thus, making the potential clinical application easier. Since M-PCN may have a malignant potential, measuring miR-200b level in cyst fluid may have a clinical importance. Although this approach should be further optimized with a larger patient cohort, our results suggest that profiling miRNA, focusing on miR-200b, may be useful in categorizing patients with pancreatic cysts.

## Methods

### Patients

The Medical Research Council of Hungary (ETT-TUKEB, IV/6522-1/2020/EKU) approved all experiments involving human samples and written informed consent was obtained from patients. All methods were performed in accordance with the relevant guidelines and regulations (World Medical Association’s Declaration of Helsinki). Inclusion of patients was decided by a multidisciplinary panel of experts. We selected adults with pancreatic cystic lesions who had the indication for EUS based on the current guidelines ^4^. Briefly, patients were selected for EUS if any of the ’worrisome features’ were present (e.g. pancreatitis in patient’s history, cystic diameter between 30 and 40 mm, enhancing mural nodule less than 5 mm in size, enhancing or thickened cystic wall, main pancreatic duct diameter of 5–9.99 mm, elevated serum CA19-9, more than 5 mm annual increase in the cystic diameter). If any of the absolute indications for surgical treatment was present, including obstructive jaundice, cystic diameter greater than 40 mm, enhancing mural nodule greater than 5 mm, main pancreatic duct diameter over 10 mm or the suspicion of the involvement of the main pancreatic duct, the patients were also selected for EUS. Patients were excluded from the study if there were not enough samples left (< 500 µL) after performing all the diagnostic tests required by the guidelines (CEA, cytology etc.). Patients were categorized into not-M and M based on multidisciplinary panel discussions. The discovery cohort had n = 7 included patients for the not-M and n = 6 for the M groups. The validation set contained n = 30 and n = 24 included persons in the not-M and M-groups, respectively. Three not-M and two M samples were excluded (all from the validation cohort) due to visible blood contamination after centrifuging the samples. Clinical and diagnostic parameters of the included patients are presented in Table S8.

### Human pancreatic cyst fluid samples

An Olympus EU-ME2 GF-UCT180 device was used to carry out the examinations and fluid samples were taken with a fine needle aspiration (FNA) technique from the pancreatic cyst fluid content of the patients (using an EchoTip^®^ Ultra Endoscopic Ultrasound Needle). Pancreas cystic fluid samples were collected in Vacuette tubes (Greiner Bio-One, 456085), centrifuged at 300*g* for 5 min and supernatants were aliquoted and frozen at − 80 °C for long-term storage (max 30 months). Aliquots were used only once for EV isolation and/or total cyst fluid analysis.

### Nanoparticle tracking analysis (NTA)

Cyst fluid samples (500 µL) were serially centrifuged at 300*g* for 5 min, at 2000*g* for 20 min and at 12,500*g* for 40 min (all steps at 16 °C). Aliquots were taken from the supernatant after each centrifugation step. Serial dilutions (50×, 100×, 500×, 1000× and 5000×) were prepared from the samples with phosphate buffered saline (PBS) and 1 mL was analyzed in NTA measurements. The particle size distribution and concentration were measured on a ZetaView Z-NTA instrument (Particle Metrix). For each sample we chose the dilution that produced EV concentration within the optimal detection limits of the instrument. According to the default settings, eleven cell positions were scanned at 25 °C to increase the reliability of the measurements. The camera settings were equal in all samples measured, as follows: auto expose, gain: 28.8, offset: 0, shutter: 100, sensitivity: 73. The videos were analyzed with a minimum area of 5, maximum area of 1000 and a minimum brightness of 20 by the ZetaView Analyze software 8.05.10. In some experiments EVs (after centrifugation steps at 300*g* and 2000*g*) were labelled with the membrane staining lipophilic BioxML-Yellow dye (Bioxol, Budapest, Hungary) ^43^. Calibration was performed with fluorescent Yellow-Green (YG) nanoparticles and parameters were set as follows: shutter: 100, sensitivity: 85, correction factor: 1.27. Percentage of fluorescently stained particles of all detected particles was calculated. To evaluate the stability of the colloidal system we measured zeta potential of the EV samples in 2 SL-positions, diluted in 0.1× PBS/pH = 6.9.

### EV detection by antibody-coated beads and flow cytometry

Samples were centrifuged at 300*g* for 5 min. 200 µL of the supernatant or its 10× dilution was incubated with 8 µL or 4 µL anti-CD63 or anti-CD81-coated magnetic beads (Thermo Fisher, 10606D and 10616D, respectively) overnight with rotation at 4 °C. Beads were then washed three times with PBS and they were re-suspended in 50 µL PBS for labelling. The percentage of positive beads was determined with anti-CD63 PE (2 µL/50 µL, SAB47000218, Merck) or anti-CD81 FITC (1 µL/50 µL, A15753, Molecular Probes) antibodies and flow cytometry (FACS Calibur, BD Biosciences). To determine the non-specific background, DMEM was incubated with the beads and these beads were labelled as controls.

### Transmission electron microscopy (TEM)

The supernatant after the last step of differential centrifuging (at 12,500*g*) was ultracentrifuged (UC) at 100,000*g* for 70 min at 4 °C. The pellet was resuspended in PBS and UC again. EVs were then fixed with 4% paraformaldehyde (PFA) for 30 min and 2% PFA + 2% glutaraldehyde for 30 min at room temperature, rinsed with PBS and post-fixed in 1% OsO_4_ for 15 min. The pellet was then dehydrated in graded ethanol, treated with 1% uranyl acetate in 50% ethanol for 30 min and embedded in Taab 812 (Aldermaston, T031). After polymerization at 60 °C for 12 h, ultrathin sections (50–60 nm) were cut with a Leica UCT ultramicrotome (Leica Microsystems, UK). TEM images were taken with a Hitachi 7100 TEM instrument (Hitachi Ltd, Japan) equipped with a Veleta 2k × 2k MegaPixel side-mounted TEM CCD camera (Olympus, Tokyo, Japan).

### miRNA analysis

500 µL of the supernatant after centrifugation at 300*g* was incubated with a mixture of 20 µL anti-CD63-coated and 10 µL anti-CD681-coated magnetic beads overnight with rotation at 4 °C. In experiments using identical initial EV numbers (7 × 10^8^ EVs determined with NTA), DMEM (Thermo Fisher) was added to the samples up to 500 µL and samples were incubated with the same amount of beads. Beads were then washed three times with PBS (3 × 500 µL) and they were lysed with 700 µL Qiazol (Qiagen). Total RNA, including miRNA, was isolated with the miRNEasy Micro Kit (Qiagen) following the manufacturer’s description. When analyzing free miRNA, 400 µL cystic fluid sample was processed with the miRNEasy serum/plasma kit (Qiagen).

For miRNA array cards, 3 µL total RNA was reverse transcribed with Megaplex RT primers and the samples were then pre-amplified with Megaplex PreAmp Primers (Thermo Fisher) according to the manufacturer’s description. TaqMan™ Array Human MicroRNA A Cards v2.0 (Thermo Fisher) were measured on an ABI 7900HT instrument. The threshold was set to 0.2. When comparing the PC and serous PCN (together: not-M PCN) with the M-PCN group, miRNAs present (Ct < 35) in all samples of a specific condition, but present in maximum one sample of the other group were chosen for further analysis.

For measuring individual miRNAs, 2 µL total RNA was reverse transcribed with the TaqMan^®^ Advanced miRNA cDNA Synthesis Kit (Thermo Fisher) according to the manufacturer’s protocol. In experiments with normalization for equal RNA amount, we used 25 ng total RNA diluted up to 2 µL in water. miRNA levels were then analyzed with the TaqMan^®^ Fast Advanced Master Mix, TaqMan^®^ Advanced miRNA Assays (Thermo Fisher) and an ABI 7900HT Fast real-time PCR instrument or a QuantStudio™ 7 Flex instrument (Thermo Fisher) with 40 cycles. The assay IDs are listed in Table S9. To obtain comparable results, measurements from the same experimental sets were carried out on the same instrument.

### Statistical analysis

Mann–Whitney U-test and chi-square test were used with *p < 0.05, **p < 0.01 and ***p < 0.005 significance levels. Microsoft Excel, and SPSS version 25 softwares were used for statistical evaluation and visualization. Mean + SD for bar graphs, or median, 25th and 75th percentiles for box plots are shown.

### Supplementary Information

Supplementary Legends.

Supplementary Table S1.

Supplementary Table S2.

Supplementary Table S3.

Supplementary Table S4.

Supplementary Table S5.

Supplementary Table S6.

Supplementary Table S7.

Supplementary Table S8.

Supplementary Table S9.

## Data Availability

Further data is available upon request by emailing the corresponding author, Dr. Zoltán Wiener.
