# Supplementary material for: MiR-200b categorizes patients into pancreas cystic lesion subgroups with different malignant potential
Source: Sci Rep. 2023 Nov 14;13:19820. doi: 10.1038/s41598-023-47129-1 (PMC10646105; doi:10.1038/s41598-023-47129-1)
Supplement: Supplementary file 1 — Supplementary Legends. [file 41598_2023_47129_MOESM1_ESM.pdf]

List of supplementary files

MiR-200b categorizes patients into pancreas cystic lesion subgroups with different malignant potential

Márton Benke[§], Anikó Zeöld[§], Ágnes Kittel, Delaram Khamari, István Hritz, Miklós Horváth, Bánk Keczer, Katalin Borka, Ákos Szücs[*], Zoltán Wiener[*]

[§]Shared first authorship, [*]shared last authorship

Table S1. Low-density miRNA array, Ct values (Ct<35)

Table S2. Ct values from the discovery samples

Table S3a-b. Ct values for the validation cohort

Table S4. Ct values when starting from an equal number of EVs

Table S5. Ct values when starting from an equal EV RNA amount

Table S6. EV miRNA analysis (volume-normalized) from operated patients

Table S7. Ct values when starting from an equal RNA amount from total cyst fluid

Table S8. Data and diagnostic values of patients with samples included in the study, Table S8b. Summary of patient data within the study groups

Table S9. TaqMan assay IDs used in our studies
